# Fake news and false memory formation in the psychology debate

**DOI:** 10.1016/j.ibneur.2023.06.002

**Published:** 2023-06-08

**Authors:** Candela S. Leon, Matías Bonilla, Luis I. Brusco, Cecilia Forcato, Facundo Urreta Benítez

**Affiliations:** a Laboratorio de Sueño y Memoria, Departamento de Ciencias de la Vida, Instituto Tecnológico de Buenos Aires (ITBA), Buenos Aires, Argentina; b Consejo Nacional de Investigaciones Científicas y Tecnológicas (CONICET), Buenos Aires, Argentina; c CENECON, Centro de Neuropsiquiatría y Neurología de la Conducta (CENECON), Buenos Aires, Argentina; d Innocence Project Argentina, Buenos Aires, Argentina

**Keywords:** Fake news, Encoding, Consolidation, Congruence effect, Disclaimer

## Abstract

Fake news can generate memory distortions and influence people's behavior. Within the framework of the great debates, the tendency to generate false memories from fake news seems to be modulated by the ideological alignment of each individual. This effect has been observed mainly around issues involving large sectors of society, but little is known about its impact on smaller-scale discussions focused on more specific populations. In this work we examine the formation of false memories from fake news in the debate between psychological currents in Argentina. For this, 326 individuals aligned to psychoanalysis (PSA) or Evidence-Based Practices (EBP) observed a series of news (12 true and 8 fabricated). The EBP group remembered or believed more fake news that damaged PSA. They also remembered with greater precision the statements of the news that harmed their own school, than those referring to others. These results could be understood as the product of an imbalance in the commitment between the different parties, since the group that proposes the paradigm shift (EBP) exhibited a congruence effect, while the group whose orientation is hegemonic in this field (PSA) did not show any effect of ideological alignment. The fact that the congruence effect is manifested to some extent in settings as relevant as the education of mental health professionals, highlights the need to move towards more careful practices in the consumption and production of media.

## Highlights

• Fake news generates false memories, especially if it is aligned with the reader.
• This “congruence effect” has only been observed in great social debates.
• We evaluated the congruence effect in the debate between psychological currents.
• The congruence effect is partially manifested in this population.
• The congruence effect is dependent on the level of commitment to the debate.

## 1 Introduction

The term *fake news* can be defined as ''false, often sensational, information spread under the guise of news reporting'' (Collins Dictionary, 2021). However, the concept of fake news can also be used for news satire, news parody, fabrication, manipulation, publicity and propaganda (Tandoc Jr et al., 2018). This term gained popularity and its use spread after Donald Trump accused the press of fabricating fake news against him, in the context of the 2016 United States election campaign (Cunha et al., 2018, Pengelly, 2017). The growth in internet reach has allowed people to access more information and it has also increased the possibilities of developing and sharing unverified information (Greifeneder et al., 2021). In recent decades, fake news had been used as a political resource against opposing ideological groups, as was observed during the COVID-19 pandemic around the effectiveness of vaccines or even the existence of the virus (Carrion-Alvarez and Tijerina-Salina, 2020).

The phenomenon of fake news has been widely analyzed from the perspective of social sciences or psychology (Pennycook and Rand, 2021; Greifeneder et al., 2021; Di Domenico et al., 2021). In the last decade several studies have found that fake news can generate false memories (Murphy et al., 2019, Greene et al., 2021). False memories are defined as the ability to recall events that never occurred (Loftus, 2003), and these memories can be of entire events or partially modified information (Newman & Lindsay, 2009). Furthermore, these false memories can be naturally generated or can be artificially induced (Loftus and Bernstein, 2005). The factors that influence their formation include the passage of time, age, proneness to dissociation, attentional capacity, ideological congruence and familiarity with memory content, among others (Fandakova et al., 2018, Heaps and Nash, 1999, Frenda et al., 2013, Murphy et al., 2019, Anaki et al., 2005, Kersten and Earles, 2017; Armaly and Enders, 2023).

In 2013, Frenda et al. observed that people were susceptible to generating false memories from fake news when they were consistent with their political ideology, a phenomenon known as congruence effect. In a similar study, Murphy et al. (2019) found that almost half (48 %) of the participants recall at least one of the two fake news presented to them, and that they were also more likely to remember them when the news were consistent with their ideology, i.e. the probability of generating false memories due to fake news exposure increased significantly when the content was consistent with the ideology. This effect was founded in political issues related to elections (Frenda et al., 2013) and referendums around important decisions for society like abortion or Brexit (Murphy et al., 2019, Greene et al., 2021), but it was also extended to debates such as feminism (Murphy et al., 2021) or the ideological position around the COVID-19 pandemic (Greene and Murphy, 2021). Thus, the congruence effect has been observed in the context of great discussions that affect general sectors of the population. However, the cognitive biases involved in the acceptance of fake news distinctively affect different groups (Preston et al., 2021), so the effect may not be replicated in particular environments.

In Argentina and various Latin American countries such as Chile or Mexico exists a particular phenomenon regarding the teaching of psychology at universities. The curriculum in most Argentine public universities is mostly oriented towards psychoanalysis 49.8 % (Freudian, post-Freudian or Lacanian) and other psychology schools have little or no place (9.1 % cognitive-constructivist and 4.9 % cognitive behavioral therapy) (Fierro, 2015). This generates several concerns in psychology students and professionals, leading to debates in social networks and academic circles around the need for a curriculum that contains the latest scientific advances throughout the world. The debate is organized around two positions, one that supports the current curriculum and ascribes to having a psychoanalytic approach in their professional practice and the other side seeks to include Evidence-Based Practices (EBP) such as cognitive and behavioral therapy (CBT) (Fierro et al., 2018, Korman, 2020, Fierro and Araujo, 2021). The main objective of this study was to assess whether the congruence effect is affecting this niche, as it does in more massive discussions.

Given the enormous health and economic implications of psychological practice, the impact of false memories generated by fake news in this debate may be critical when reaching a positive conclusion that benefits both the discipline and society in which it is immersed. For this reason, it is essential to direct the attention of the participants to the factors that can influence their position, and generate cognitive distortions. There are effective strategies that the media can adopt to avoid misinformation, such as explicit disclaimer about possible fake news (Ecker et al., 2010).

Thus, we carried out an online experiment in a population of argentine psychology students and graduates. Our hypothesis was that the congruence effect is also generated in this specific population. We expect participants to report more false memories for fake news consistent with their theoretical position.

Additionally, we carried out two exploratory analyses aimed to describe in greater depth the phenomenon of false memories from fake news. Contextual details of memory tend to decline with time, and the power of a stimulus to generate false memories increases when its circumstances of encoding are weakened leading to source-monitoring errors. Thus, we further analyzed how ideological congruence can modulate the long-term memory for fake news text's. Finally, we evaluated the effect of a disclaimer a week after the initial exposition to the fake news on an immediate re-exposition.

## 2 Experimental procedures

### 2.1 Participants

The participants (*N* = 326) were psychology students and graduates of argentine universities. The sample size was decided according to previous studies sharing similar designs (Mangiulli et al., 2022, Lee et al., 2020) They were recruited through advertisements on social networks. Prior to their participation, they read and agreed to the informed consent approved by the Alberto Taquini Biomedical Research Ethics Committee. Ages ranged from 18 to 56 years (27.65 ± 7.22) of which 93.6 % were between the ages of 18 % and 40 % and 6.4 % between the ages of 41 and 56. All experiments were carried out on online platforms. There were 26 participants who did not define a preferred theoretical framework or had an ambiguous orientation and were therefore excluded from the experiment (*N* = 300). Then, 130 of the initial participants decided not to participate in the second session or failed to follow the schedule. Therefore, for the Cue recall task, there were 170 participants. The Revision Task was completed by 136 participants, of which 63 previously received the disclaimer on the possibility of having seen fake news and 73 did not receive any warning.

### 2.2 Procedure

**Fig. 1:**
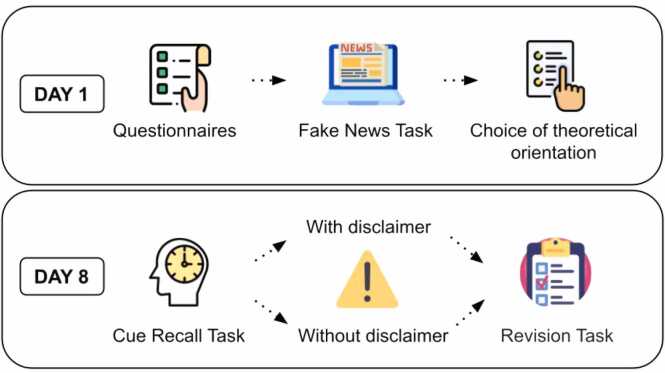
.Experimental procedure. The procedure was divided into two days: on day 1 the participants completed the Fake News Task. One week later, they completed the Cue recall task. Finally, they had to carry out a Revision task. Icons ''Choose'', ''Options'', ''Clock'', ''Journal'', ''Laptop'', ''Test'', ''Warning'' and ''Task List'' ' were created by Freepik [https://www.flaticon.com/authors/ freepik] from www. flaticon.com.

The experiment was divided into two days. On the first day, the participants received a link to access the online form. They provided their consent to participate in the experiment, completed the sociodemographic questionnaire and the Fake News Task. At the end of this day, the participants were asked to express their level of alignment with each psychology school, through a grid that allowed them to distribute 100 points among all currents. The experimental groups were established on the basis of this choice. The participants were divided in "Evidence-Based Practices" group (*N* = 216, 182 women, 33 men; mean age ± SD: 27.40 ± 7.01) and the "Psychoanalysis" group (*N* = 84, 71 women, 13 men; mean age ± SD: 28.65 ± 7.58). 7 days later, the participants received a second link and the Cue recall task was presented. After finishing it, the subjects were divided into two groups, and one of them received a disclaimer, alerting them to the possibility of having been exposed to fake news. At that point, all participants completed the Revision task, having the opportunity to review their choices from day 1 and keep or change them. Before finishing, all subjects received information about the study and were notified of the presence of fake news (Fig. 1).

### 2.3 Sociodemographic questionnaire

Collects data referring to the age and gender of the participants, as well as their position within the psychology career and the institution in which they graduated or are currently studying.

### 2.4 Stimuli

**Fig. 2:**
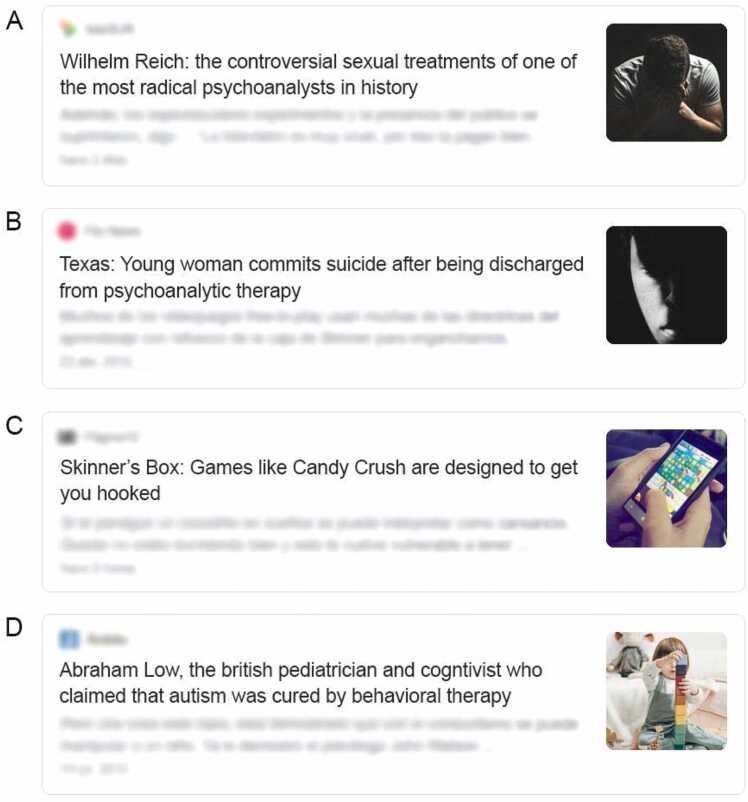
True and fake news. A. True news item against psychoanalysis. B. Fabricated new item against psychoanalysis. C. True news item against evidence-based practices. D. Fabricated news item against evidence-based practices.

The initial group of stimuli consisted of 52 news items, formed by a headline accompanied by an image, following a format similar to Google Feed (Fig. 2) to increase the feeling of truth (Strange et al., 2011). 22 of those items were true news (collected from the web) and 30 were fabricated for the experiment. This first group of news went through a selection process in order to obtain a smaller set of clearly polarized, highly realistic news. For this, a control study (*N* = 40) was conducted, in which the participants should tell whether each news was real (taken from the web) or made up by the experimenters. Additionally they were asked for the alignment of each item (which psychology school was being criticized). The resulting set was composed of 20 news articles (Supplementary material), 12 were true news (extracted from the media) and 8 were fake news, that is, fabricated for this experiment. 4 of those fake news were detrimental to one of the theoretical currents and 4 were detrimental to the other. In addition, in order to control that the stories did not differ in salience, a counterbalancing was carried out between the fake news. For this, a second set of 8 fake news was created, mirroring the first. These items contained identical images and stories, but the text was slightly modified to target the opposite current. Participants were randomly assigned to one of the two sets.

### 2.5 Tasks

#### 2.5.1 Fake news task

The news appeared randomly on a monitor’s screen and participants had to choose within 30 s one of the following options: “I remember seeing/hearing this”, “I don't remember seeing/hearing this but it happened”, “I remember this differently”, “I don't remember it”. For operational purposes, in the cases in which we presented fake news, the response “I remember seeing/hearing this” was taken as a measure of false memory and the response “I don't remember seeing/hearing this but it happened” was taken as a measure of false belief. While the rest of the answers (“I remember this differently”, “I don't remember it”) were not counted. The division between memory and belief was made by previous recommendations (see Wade et al., 2018). In the case of true news, only the response “I remember seeing/hearing this” was taken as a measure of true memory.

#### 2.5.2 Cue recall task

Participants had to observe the same stimuli of the Fake news task composed of image plus headline, with the difference that the headlines were partially blurred. They had to complete the blurred parts with the correct sentences, based on their memories of the previous task. In all cases, the blurred part was the gist of the news. The order of the news was randomized. This task was included as a measure of the memory strength, since stronger memories are usually related to better source monitoring, that is, greater accuracy in determining whether the news being presented are new or previously seen (Johnson et al., 1993, Zaragoza and Mitchell, 1996).

#### 2.5.3 Disclaimer

Participants read a paragraph that warns them of the possibility that some of the news they were exposed to, were fabrications created by the researchers with deception purposes.

#### 2.5.4 Revision task

Half of the participants previously received the disclaimer and the other did not, and then, were asked to observe the news articles they watched the previous week and had the possibility to repeat or modify their choices from day 1. The response options were the same as in the Fake news task: “I remember seeing/hearing this”, “I don't remember seeing/hearing this but it happened”, “I remember this differently”, “I don't remember it”. The order of the news was randomized.

### 2.6 Statistical analysis

Statistical analyzes were performed using the IBM SPSS Statistics 25 software. The overall distribution was not normal, therefore non-parametric tests were used to compare the performance of the groups.

#### 2.6.1 Fake news task

Initially the 8 fake news were observed: To analyze the false memories generated by the participants, we scored the response “I remember seeing/hearing this” as positive, while the rest were negative. To analyze the congruence effect, both the response "I don't remember having seen/heard this, but it happened" and "I remember seeing/hearing this" were taken as positive, and the other two as negative. In addition, the 12 true news were analyzed in order to control the baseline level of information that participants had. For this, the answer "I remember seeing/hearing this" was counted. We compared the performance of the groups using the two tailed Mann–Whitney test. This was done for both types of fake news, that is, for fake news that criticized psychoanalysis, and the same for fake news that criticized Evidence-Based Practices. For this analysis, cases that did not have a defined position around their theoretical orientation were excluded. In addition, the true news were analyzed in order to control the baseline level of information that the participants had.

#### 2.6.2 Cue recall task

Three independent evaluators judged whether the person had answered correctly with the gist of the sentence. If two or more agreed, the answer was considered valid, and a point was given. If only one or none considered it correct, no points were assigned. One of the fake news was not properly displayed to the participants, so it was removed from the analysis. To assess whether ideological congruence can modulate the long-term memory of the text of fake news, a two tailed Mann-Whitney U test was used. The performance of the groups in each set of fake news was compared, that is, for the set of fake news that harmed psychoanalysis and for the set of fake news that harmed EBP.

#### 2.6.3 Disclaimer

To weigh the effect of the disclaimer on the generation of false memories, the number of false memories reported in the Revision task was compared between the groups with and without disclaimer, using a two tailed Mann-Whitney U test. Also, the change in the number of false memories reported between the Fake news Task and the Revision task was observed, both for the group that received disclaimer and for the group that did not. To perform this analysis, the Wilcoxon Signed-Ranks test was carried out.

The raw data supporting the conclusions of this article is available in https://zenodo.org/record/7600219#. Y9vmaXbMI2w.

All tests were performed with a fixed alpha of 5 %.

## 3 Results

### 3.1 Fake news task

**Fig. 3:**
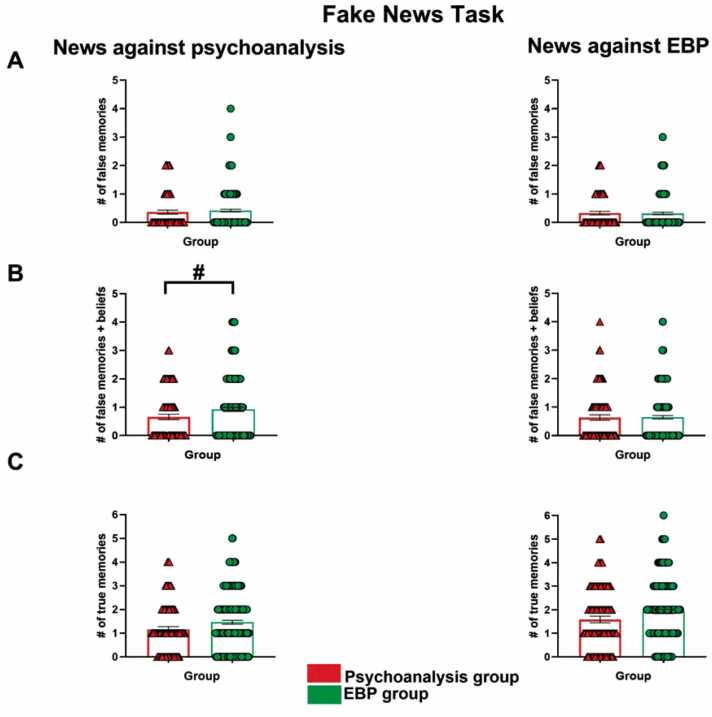
Fake News Task. A. Number of false memories ± SEM. B. Number of false memories and beliefs ± SEM. C. Number of true memories ± SEM. #, *p* = 0.06.

First, it was checked that the groups did not differ in credulity level. Both showed similar rates of acceptance of recall and belief in both true and fake news (Psychoanalysis group: Mdn = 2.00, EBP group, Mdn = 3.00, U = 8085.50, z = −1.56, *p* = 0.11, r = −0.09). To verify that both groups have the same level of prior information, the responses obtained regarding true news were compared. There were no differences for recalls of true news that harms psychoanalysis (Psychoanalysis group: Mdn = 0.00, EBP group, Mdn = 0.00, U = 8481.50, z = −1.07, *p* = 0.28, r = −0.06. Fig. 3C) nor for the true news that harmed Evidence-Based Practices (Psychoanalysis group: Mdn = 1.03, EBP group, Mdn = 1.00, U = 8624.00, z = −0.81, *p* = 0.41, r = −0.04. Fig. 3C).

We found a trend that indicates that the EBP group had more recalls and beliefs (i.e. significantly more responses of “I remember seeing/hearing this” and "I don't remember having seen/heard this, but it happened") of fake news perjudicial to psychoanalysis, than the Psychoanalysis group (Psychoanalysis group: Mdn = 0.00, EBP group, Mdn = 0.00, U = 7983.00, z = −1.84, *p* = 0.06, r = −0.10. Fig. 3B). However, no differences were observed for recalls and beliefs against EBP (Psychoanalysis group: Mdn = 0.00, EBP group, Mdn = 0.00, U = 9087.00, z = −0.08, *p* = 0.93, r = −0.004. Fig. 3B). There were no differences for the fake news recall against psychoanalysis (Psychoanalysis group: Mdn = 0.00, EBP group, Mdn = 0.00, U = 8626.00, z = −0.93, *p* = 0.34, r = −0.05. Fig. 3A), neither for fake news recall against Evidence-Based Practices (Psychoanalysis group: Mdn = 0.00, EBP group, Mdn = 0.00, U = 8854.00, z = −0.55, *p* = 0.41, r = −0.03. Fig. 3A). Finally, observing the beliefs separately, there are no differences between the groups, neither in news that harms psychoanalysis (Psychoanalysis group: Mdn = 0.00, EBP group, Mdn = 0.00, U = 9114.50, z = −0.04, *p* = 0.97, r = −0.002) nor in news that harms evidence-based practices (Psychoanalysis group: Mdn = 0.00, EBP group, Mdn = 0.00, U = 8329.00, z = −1.48, *p* = 0.13, r = −0.08).

### 3.2 Exploratory analysis

#### 3.2.1 Cue recall task

**Fig. 4:**
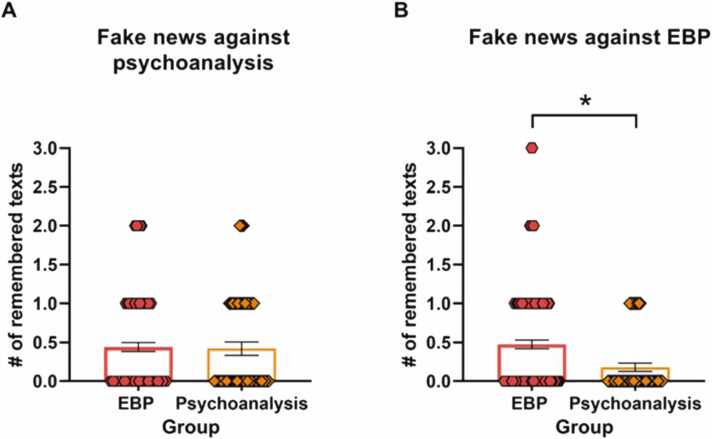
Cue recall task. A. Number of gists remembered of fake news against psychoanalysis ± SEM. B. Number of gists remembered of fake news against EBP ± SEM. * , *p* < 0.05.

The performance of the groups in each set of fake news was compared using the Mann-Whitney test. There were no differences in the persistence of texts of fake news that harmed psychoanalysis (Psychoanalysis group: Mdn = 0.00, EBP group, Mdn = 0.00,U= 2965, z = −0,14, *p* = 0.89. Fig. 4A). In contrast, there were significant differences in the persistence of texts of fake news that were against Evidence-Based Practices, since the EBP group remembered more texts that attacked their own orientation (Psychoanalysis group: Mdn = 0.00, EBP group, Mdn = 0.00, U= 2263, z = −3.03, *p* = 0.002, r = −0.23. Fig. 4B).

#### 3.2.2 Disclaimer

**Fig. 5:**
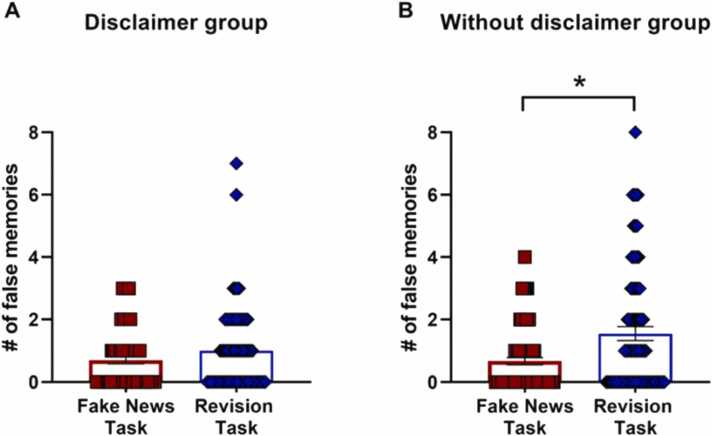
Disclaimer effect. A. Number of false memories reported in the Fake news task and the Revision task for Disclaimer group ± SEM. B. Number of false memories reported in the Fake news task and the Revision task for Without disclaimer group ± SEM.

About the effect of the disclaimer on the number of false memories reported during the Revision task, no significant differences were found between the groups (Without disclaimer group: Mdn = 1.00, Disclaimer group, Mdn = 1.00, z = −1.20, *p* = 0.23.). Furthermore, for the Without disclaimer group the number of false memories reported on the Revision task (day 8) significantly increased compared to those reported on the Fake news task (day 1) (Wilcoxon test, day 8: Mdn = 1.00, day 1: Mdn = 0.00, z = −3.59, *p* < 0.00, r = −0.29. Fig. 5B). In contrast, the Disclaimer group showed no significant change in the number of false memories between the Fake News Task and the Revision task, (day 8: Mdn = 0.00, day 1: Mdn = 1.00, z = −1.79, *p* = 0.07. Fig. 5A).

### 3.3 Control measures

The groups were equitable in terms of gender (EBP group: Females (182), Males (33); Psychoanalysis group: Females (71), Males (13) [*χ*2(2) = 0.97, *p* = 0.56), the age (EBP group: 27.40 ± 7.01, Psychoanalysis group: 28.65 ± 7.58, t (299) = −1.323, *p* = 0.18), the level of consumption of online news (EBP group: Always (38), Sometimes (142), Never (36); Psychoanalysis group: Always (11), Sometimes (59), Never (14) [χ2(2) = 0.88, *p* = 0.64) or for the stage of the psychology degree (EBP group: First half (80), Second half (70), Graduate (66); Psychoanalysis group: First half (24), Second half (26), Graduate (34) [*χ*2(2) = 1.16, *p* = 0.55).

## 4 Discussion

In the present study, we mainly investigate how ideological congruence can modulate fake news processing in the context of the theoretical debate of psychology in Argentina. Contrary to what we expected, people did not differ in recalls of fake news that harmed the opposite group. However, when belief and memory responses were analyzed together, the congruence effect was present in the EBP group for fake news from the opposing side. Although, this difference was not observed for fake news that damaged Evidence-Based Practices.

Regarding cue recall task, where the persistence of the news text was evaluated, we observed that the EBP group had significantly more persistence of information related to their own group, even taking into account that the news were detrimental to its theoretical orientation.

Finally, regarding the beneficial effect of the disclaimer, we observed that no difference could be seen in the revision task, but it was revealed in the reduction of false memories between the Fake News Task (day 1) and the Revision Task (day 8) in the Disclaimer group.

The results of the main task suggest that the biased vision effect occurs for one of the groups. A possible explanation for this differential effect could be the level of engagement that the groups have with the debate. Previous studies found that people generated more false memories when it came to topics of great interest, regardless of the level of knowledge (O’ Connel & Greene 2017). This could indicate that, despite the fact that the debate between positions is powerful and generates strong discussions, it probably does not reach a sufficient level of massiveness in both groups.

The PBE group also remembered significantly more the headlines of the news that addressed to the group itself. There is a certain consensus around the idea that the emotion evoked by a piece of information tends to condition the strength of the memory built based on it (Bradley et al., 2007). The fact that the EBP group remembers the statements that attack their position with more intensity, aligns with this idea. On the contrary, the PSA group does not distinctly remember any type of statement, creating an apparent contradiction. Such a contradiction could be just another consequence of the disparity in the level of involvement between both groups, since an individual who does not consider himself part of a conflict will be less aroused when receiving criticism.

The results obtained in the disclaimer task go in line with previous findings indicating that inoculation maneuvers have positive effects in reducing damage caused by the dissemination of fake news (Lewandowsky and Van der Linden, 2021; Pennycook and Rand, 2019).

Within the limitations of this work, is the fact of not having controlled the level of interest in the debate that the participants actually had. Another limitation was the disproportionate number of participants in each group and a possible explanation for this phenomenon could be the lack of interest in scientific studies of the group related to psychoanalysis, despite being the majority group in the academic field of psychology in Argentina.

In the future, it would be interesting to evaluate how analytical thinking and cognitive ability influence the processing of this kind of news in this type of population, since it has been previously observed that engaging in analytical thinking is a predictor of resistance to false memories (Pennycook and Rand, 2019, Greene et al., 2021), as well as lower cognitive ability was associated with an increased effect of ideological congruence on false memories (Murphy et al., 2019).

In sum, it was not observed that the congruence effect modifies the rate of false memories. However, it was observed that this population makes decisions guided by ideological congruence. This is concerning because it suggests that people make decisions based on bias even in very specific settings that are not tied to big political discussions or deep civil debates. Finally, we observe that alerting people to the possible existence of fake news can counteract the cumulative effect of false memories that can be generated by the repetition of fake news. This strategy has become quite popular and is used in different media, for example Twitter (Hartwig and Reuter, 2019), and has proven to be a way to combat fake news in the short term, but recent work indicates that it helps briefly and has limited reach (Grady et al., 2021).

## Funding

This work was supported by AGENCIA PICT Serie A N°02666 to CF.

## Financial disclosure

The funders had no role in study design, data collection and analysis, decision to publish, or preparation of the manuscript. The authors have declared that no competing interests exist.

## Ethical statement

I have read and have abided by the statement of ethical standards for manuscripts submitted to IBRO Neuroscience Reports..

## CRediT authorship contribution statement

**Candela S. Leon:** Conceptualization, Methodology, Investigation, Formal analysis, Writing – original draft, Writing – review & editing. **Matías Bonilla:** Conceptualization, Methodology, Investigation, Writing – review & editing. **Luis I. Brusco:** Writing – review & editing. **Cecilia Forcato:** Conceptualization, Methodology, Writing – original draft, Writing – review & editing**. Facundo Urreta Benítez:** Conceptualization, Methodology, Investigation, Formal analysis, Writing – original draft, Writing – review & editing.

## Author contributions

**CSL, MB, FAUB** and **CF** made substantial contributions to the conception and design of the work. **CSL, MB** and **FAUB** ran the experiments. **CSL** and **FAUB** performed the statistical analyses. **CSL, FAUB** and **CF** contributed by drafting the work. **CSL, MB, FAUB, BIL** and **CF** contributed to revising it critically.

## Conflicts of Interest

The authors declare that the research was conducted in the absence of any commercialor financial relationships that could be construed as a potential conflict of interest.
