# Supplementary material for: Fake news and false memory formation in the psychology debate
Source: IBRO Neurosci Rep. 2023 Jun 8;15:24–30. doi: 10.1016/j.ibneur.2023.06.002 (PMC10285207; doi:10.1016/j.ibneur.2023.06.002)
Supplement: Supplementary file 1 — Supplementary material [file mmc1.docx]

|  | **Supplementary material** |  |  |  |  |
| --- | --- | --- | --- | --- | --- |
|  | **True News** | 1* | 2 | 3 | 4 |
| 1 | Drug agencies are an invention of neoliberal capitalism in the 1990s | 29.9** | 12.5 | 11.3 | 46.4 |
| 2 | Mario Bunge: psychoanalysis and other pseudosciences are harmful | 38.0 | 9.9 | 10.1 | 42.0 |
| 3 | A pandemic of adjusting and behavioral neoliberalism in education | 16.2 | 10.7 | 15.9 | 57.1 |
| 4 | Scientists explain why dreams have no hidden meanings | 30.4 | 18.0 | 13.3 | 38.3 |
| 5 | Little Albert, a cruel experiment on an 11-month-old baby to test phobias | 52.8 | 11.9 | 11.3 | 24,1 |
| 6 | The community gathers signatures against public psychoanalysis therapies in cases of autism | 9.0 | 9.9 | 7.5 | 73.6 |
| 7 | Skinner's Box: Games like Candy Crush are designed to get you hooked | 26.7 | 11.6 | 15.9 | 45.8 |
| 8 | Wilhelm Reich: the controversial sexual treatments of one of the most radical psychoanalysts in history | 5.2 | 7.5 | 8.4 | 78.8 |
| 9 | The psychiatrist who applied electroshocks to homosexuals | 33.0 | 23.8 | 15.4 | 27.8 |
| 1 | The feminist who denied Freud and his penis envy | 34.2 | 18.3 | 10.4 | 37.1 |
| 11 | Experts call for a review of current diagnostic methods for bipolar disorder | 19.7 | 16.5 | 7.8 | 55.9 |
| 12 | The story of Freud's Argentinian nephew: he is a psychoanalyst and questions the idea of ​​being trans before puberty | 18.8 | 4.6 | 11.0 | 65.5 |
|  |  |  |  |  |  |
|  |  |  |  |  |  |
|  | **Fake news set 1** | 1 | 2 | 3 | 4 |
| 1 | The Freudian therapist who hypnotized patients with electric shocks will go to trial | 9.9 | 7.4 | 6.2 | 76.5 |
| 2 | Abraham Low, the British pediatrician and cognitivist who claimed that autism was cured by behavioral therapy | 6.8 | 6.2 | 12.3 | 74.7 |
| 3 | Horror in Formosa: the young woman hospitalized for starvation, closed the refrigerator with a padlock as part of her psychoanalytic therapy | 8.0 | 7.4 | 8.6 | 75.9 |
| 4 | Malpractice is being investigated: He had been depressed for 5 years and his cognitive therapist refused to give him a diagnosis | 4.9 | 9.9 | 6.8 | 78.4 |
| 5 | Texas: Young woman commits suicide after being discharged from psychoanalytic therapy | 5.6 | 9.3 | 4.3 | 80.9 |
| 6 | License suspended for cognitive therapist who undressed patients to help them connect with their bodies | 7.4 | 6.8 | 10.5 | 75.3 |
| 7 | A shooter in Dakota city: "had passed all the projective techniques, he was a normal person" | 20.4 | 12.3 | 16.7 | 50.6 |
| 8 | Recent neuroimaging findings debunk Watson's concept of 'conditioning'' | 8.0 | 6.2 | 8.6 | 77.2 |
|  |  |  |  |  |  |
|  |  |  |  |  |  |
|  | **Fake news set 2** | 1 | 2 | 3 | 4 |
| 1 | The cognitive therapist who trained patients with electric shocks will go to trial | 9.3 | 12.6 | 4.4 | 73.8 |
| 2 | Donald Winnicott, the British pediatrician, and psychoanalyst who claimed that autism was cured by hypnosis | 6.6 | 9.8 | 15.3 | 68.3 |
| 3 | Horror in Formosa: the young woman hospitalized for starvation closed the refrigerator with a padlock as part of her cognitive therapy | 7.1 | 3.3 | 9.3 | 80.3 |
| 4 | Malpractice is being investigated: He had been depressed for 5 years and his psychoanalyst therapist refused to give him a diagnosis | 15.3 | 18.0 | 10.9 | 55.7 |
| 5 | Texas: Young woman commits suicide after being discharged from brief cognitive therapy | 3.3 | 6.0 | 5.5 | 85.2 |
| 6 | License suspended for psychoanalyst who undressed patients to help them connect with their bodies | 8.7 | 12.0 | 10.4 | 68.9 |
| 7 | A shooter in Dakota city: "had passed all the psychometric techniques, he was a normal person" | 15.8 | 16.4 | 20.2 | 47.5 |
| 8 | Recent neuroimaging findings debunk Freud's concept of 'superego' | 10.4 | 13.1 | 7.1 | 69.4 |
